# Environmental Lead Exposure Accelerates Progressive Diabetic Nephropathy in Type II Diabetic Patients

**DOI:** 10.1155/2013/742545

**Published:** 2013-02-28

**Authors:** Wen-Hung Huang, Ja-Liang Lin, Dan-Tzu Lin-Tan, Ching-Wei Hsu, Kuan-Hsing Chen, Tzung-Hai Yen

**Affiliations:** Department of Nephrology and Division of Clinical Toxicology, Chang Gung Memorial Hospital, Linkou Medical Center, Chang Gung University and School of Medicine, 199 Tung-Hwa North Road, Taipei 10548, Taiwan

## Abstract

Whether environmental lead exposure has a long-term effect on progressive diabetic nephropathy in type II diabetic patients remains unclear. A total of 107 type II diabetic patients with stage 3 diabetic nephropathy (estimated glomerular filtration rate (eGFR) range, 30–60 mL/min/1.73 m^2^) with normal body lead burden (BLB) (<600 **μ**g/72 hr in EDTA mobilization tests) and no history of exposure to lead were prospectively followed for 2 years. Patients were divided into high-normal BLB (>80 **μ**g) and low-normal BLB (<80 **μ**g) groups. The primary outcome was a 2-fold increase in the initial creatinine levels, long-term dialysis, or death. The secondary outcome was a change in eGFR over time. Forty-five patients reached the primary outcome within 2 years. Although there were no differences in baseline data and renal function, progressive nephropathy was slower in the low-normal BLB group than that in the high-normal BLB group. During the study period, we demonstrated that each 100 **μ**g increment in BLB and each 10 **μ**g increment in blood lead levels could decrease GFR by 2.2 mL/min/1.72 m^2^ and 3.0 mL/min/1.72 m^2^ (*P* = 0.005), respectively, as estimated by generalized equations. Moreover, BLB was associated with increased risk of achieving primary outcome. Environmental exposure to lead may have a long-term effect on progressive diabetic nephropathy in type II diabetic patients.

## 1. Introduction

 Over the past 25 years, the prevalence of type II diabetes in the USA has almost doubled, with 3- to 5-fold increases in developing countries [1]. Diabetes is now the major cause of end-stage renal disease and the primary diagnosis causing kidney disease in 20–40% of patients starting treatment for end-stage renal disease worldwide [2, 3]. However, few studies have investigated the relationship between environmental exposure to lead and diabetic nephropathy.

 Previous epidemiological studies [4–6] showed that blood lead levels (BLL) are related to renal function [4, 5] and exacerbated age-related decreases in renal function [6] in the general population, suggesting that environmental exposure to lead influences renal function in healthy individuals. Because BLL only indicates recent lead exposure [4, 7], body lead burden (BLB) is usually assessed by X-ray fluorescence to determine bone lead content and calcium disodium ethylenediaminetetraacetic acid (EDTA) mobilization tests [7]. A BLB greater than 600 *μ*g, as determined by EDTA mobilization tests, is considered lead poisoning. Previous investigations that used EDTA mobilization tests to assess BLB in nondiabetic chronic kidney disease (CKD) patients with normal BLB [8–12] suggested that environmental lead exposure is associated with progressive CKD.

A 6-year study [13] indicated that bone lead content is related to progressive elevation of serum creatinine in persons with diabetes. However, these values were not adjusted for daily urinary protein excretion or daily protein intake. A short-term 1-year observational study [14] of type II diabetic patients with diabetic nephropathy suggested that environmental lead exposure might influence progressive diabetic nephropathy. However, the observation period was too short to demonstrate the long-term toxic effect of environmental lead exposure; therefore, the estimated glomerular filtration rate (eGFR) was calculated from the American Modification of Diet in Renal Disease (MDRD) formula for CKD patients [15] rather than the Chinese formula for type II diabetic patients [16]. Hence, the relationship between low-level environmental exposure to lead and progressive diabetic nephropathy remains unclear. This 2-year prospective study was performed to clarify the relationship in type II diabetic patients.

## 2. Study Population and Methods

### 2.1. Subjects

The Institutional Review Board Committee of Chang Gung Memorial Hospital approved the study protocol. Each patient provided written informed consent.

Patients aged from 30 to 83 years who had type II diabetes mellitus with nephropathy and who received followup care at Chang Gung Memorial for more than 1 year were eligible for inclusion in this study if they met all the following criteria [14]: abnormal serum creatinine (>1.4 mg/dL); stage 3 CKD (eGFR between 30 mL/min/1.73 m^2^ and 60 mL/min/1.73 m^2^); diabetic retinopathy treated with or without laser therapy; daily urinary protein excretion of more than 0.5 g/day; no microhematuria in urine tests; normal-sized kidneys as determined by echograms; history of diabetes for more than 5 years; no known history of exposure to lead or other heavy metals; and a BLB of less than 600 *μ*g as measured by EDTA mobilization testing and 72-hour urine collection. Diabetic nephropathy diagnoses were also based on renal histological examination findings in cases where renal biopsies were performed.

The exclusion criteria were as follows: type I diabetes; renal insufficiency with a potentially reversible cause such as malignant hypertension, urinary tract infection, hypercalcemia, or drug-induced nephrotoxic effects; presence of other systemic diseases such as connective tissue diseases; use of drugs that might alter the course of renal disease such as nonsteroidal anti-inflammatory agents, steroids, immunosuppressive drugs, or Chinese herbal drugs; having joined a previous study [14]; drug allergies; and the absence of informed consent. The blood pressure of each patient was maintained at less than 140/90 mm Hg with diuretics and angiotensin-converting-enzyme inhibitors (ACEI) or angiotensin II receptor antagonists (ARA), with or without calcium-blocking agents and/or vasodilators [17]. Calcium carbonate was employed to maintain patients' phosphate levels. No patients received vitamin D3 supplements because their parathyroid hormone was below 200 pg/mL. Each patient received dietary consultation. A diabetic diet (35 Kcal/kg of body weight per day) with normal-protein intake (0.8–1.0 g of high biological value protein per kilogram of body weight per day) was recommended to each patient. A nutritionist reviewed the dietary intake of each patient every 3 to 6 months. A 24-hour urea excretion analysis was performed every 3 months to determine nitrogen balance and dietary compliance [18].

### 2.2. Measurements of Blood Lead Levels and Body Lead Burdens

BLL and BLB were measured as described previously [7–12]. BLB was measured using EDTA mobilization tests as modified by Behringer et al. [19]. Urinary excretion measured 72 hours after the intravenous infusion of 1 g of calcium disodium EDTA (Abbott Laboratories, North Chicago, IL, USA) was used to measure BLB. Blood and urine lead levels were determined by electrothermal atomic-absorption spectrometry (SpectrAA-200Z; Varian, CA, USA) with Zeeman background correction and a L'vov platform. Both internal and external quality-control procedures were applied throughout this study and achieved consistently satisfactory results. A certified commercially prepared product (Seronorm Trace Elements, Sero AS, Billingstad, Norway) was utilized to monitor intrabatch accuracy and ensure interbatch standardization. The coefficient of variation for lead measurement was <5.3%. The detection limit was 0.01 *μ*g/dL. External quality control was maintained via participation in the governmental National Quality-Control Program. Low-normal BLB was defined as <80 *μ*g and high-normal BLB was defined as >80 *μ*g and <600 *μ*g [9–12, 14]. 

### 2.3. Study Protocol

**Figure 1:**
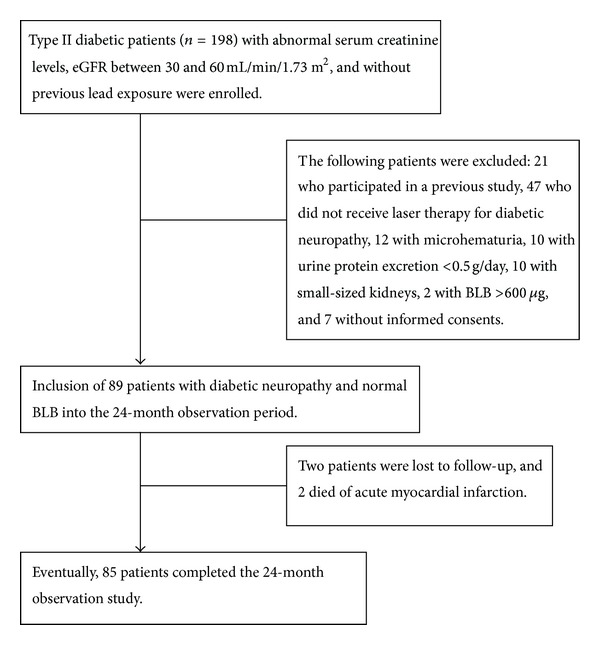
Flow chart showing the enrollment and status of patients.

Serum creatinine, glycosylated hemoglobin (HbA1c), daily urine protein excretion, daily protein intake, mean arterial pressure, cholesterol, and triglyceride levels were measured with an autoanalyzer system (model 736; Hitachi, Tokyo, Japan) at the beginning and end of the study and every 3 months during the 24-month clinical observation period. Blood pressure and body mass index were also measured at 3-month intervals. At the end of this period, we compared the changes in renal function between the 2 groups and assessed the relationship between BLB and the progressive decline of diabetic nephropathy. Renal function was assessed by creatinine clearance and eGFR (both in mL/min/1.73 m^2^ of body surface area). A modified eGFR equation for Chinese patients with type II diabetes was employed [16] (D-GFR) (mL/min/1.73 m^2^) (*R* ^2^ = 0.95): 313 × (age)^−0.494^ (years) × [SCr]^−1.059^ (mg/dL) × [Alb]^+0.485^ (g/dL) for men, and 783 × (age)^−0.489^ (years)  × [SCr]^−0.877^ (mg/dL) × [SUN]^−0.150^ (mg/dL) for women. A total of 85 patients completed the initial study period (Figure 1).

### 2.4. Outcome Measures

The primary endpoint was a 2-fold elevation in serum creatinine (measured twice, 1 month apart) from baseline values, need for long-term dialysis, or death during the 24-month observation period. The secondary endpoint was temporal changes in renal function during the study period.

### 2.5. Statistical Analysis

The differences in variables and renal function between the 2 groups were analyzed by the Chi-square test and Student's  *t*-test. All  *P*  values were two-tailed, and all results are presented as means ± SD. The Mann-Whitney  *U*  test was employed for data not normally distributed. We performed a sensitivity analysis that assigned the mean eGFR value of the treatment group to controls lost to followup and assigned the mean eGFR value of the control group to treated patients lost to followup. Generalized estimating equations (GEE) with linear analysis were employed in longitudinal multivariate analyses using SAS statistical software (version 6.12) to further assess the temporal changes in variables and associations with progressive renal function (eGFR) during the observation period. Moreover, multivariate Cox analyses were used to determine the significance of the baseline variables for predicting the primary endpoint during the study period. These models included all variables identified in the literature as related to the progression of diabetic nephropathy [12–16]. A value of  *P* < 0.05 was considered statistically significant. Data were analyzed using SPSS, version 18.0 for Windows 95 (SPSS Inc., Chicago, IL).

## 3. Results

### 3.1. Study Subjects

A total of 89 patients participated in the study and 85 completed the 24-month observation period (58 men and 31 women) (Figure 1). The following baseline data were obtained: patient mean age, 60.1 ± 9.5 years (range, 33–83); body-mass index (weight in kilograms divided by the square of height in meters), 24.9 ± 3.3 (range, 14.9–33.4); serum creatinine level, 1.9 ± 0.3 mg/dL (range, 1.5–2.8 mg/dL); eGFR, 41.3 ± 6.9 mL/min/1.73 m^2^ of body surface area (range, 30.3–59.9 mL/min/1.73 m^2^ of body surface area); daily protein excretion, 3.0 ± 2.5 g (range, 0.5–12.2 g); daily protein intake, 0.97 ± 0.18 g/kg (range, 0.58–1.63 g/kg); HbA1c, 8.3 ± 1.9% (range, 5.7–14.7%); BLL, 4.3 ±  1.1 *μ*g/dL (range, 0.8–10.4 *μ*g/dL); and BLB, 109.9 ± 52.3 *μ*g (range, 14.4–316.8 *μ*g). Sixty-two patients (70.0%) had hyperlipidemia. Eighty-four patients (95.5%) had hypertension, and they were treated with ACEI or ARA. Fourteen patients (15.7%) smoked. Seventy-six patients (85.4%) had retinopathy, which was treated with laser therapy. Among all the study patients, 29 (32.6%) had a history of cardiovascular diseases, including myocardial infarction, congestive heart failure, stroke, and diabetic foot. BLL was associated with BLB in all study patients (*r* = 0.274,  *P* = 0.009).

### 3.2. Longitudinal Followup for a 24-Month Period

Table 1 summarizes demographic data, baseline chronic disease condition, use of ACEI or ARA, daily urinary urea and protein levels, and body lead burden for participants in each group. No significant differences in these baseline values were noted between the 2 groups on initial assessment or during the observation period.  Table 2 compares the progression of diabetic nephropathy between the high-normal BLB and low-normal BLB groups during the observation period. Creatinine clearance and eGFR were higher in the low-normal BLB group than in the high-normal BLB group during months 18 to 24 of the observation period. Similar results were obtained in the sensitivity test (Table 3).

### 3.3. Outcome Measures

**Figure 2:**
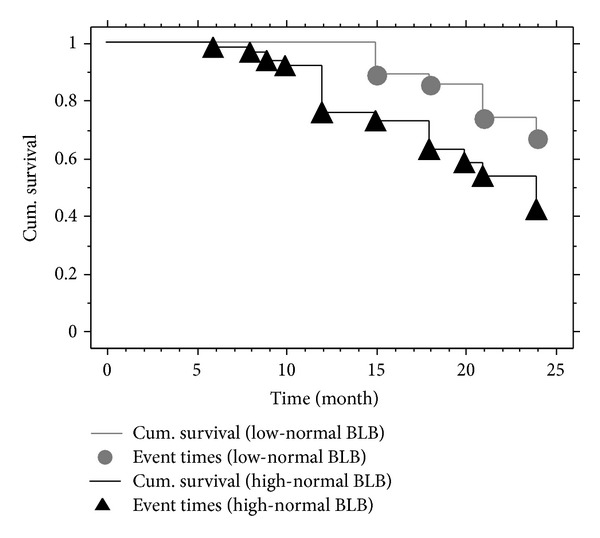
Kaplan-Meier analysis showing that patients with high body lead burden (BLB) (>80 and <600 *μ*g) had a higher likelihood (58.1%, 36/62) of achieving the primary endpoint than those with low BLB (<80 *μ*g) (33.3%, 9/27; Logrank tests, Chi-square = 5.17,  *P* = 0.023) during the 24-month followup period.

Thirty-nine patients had a 2-fold elevation in serum creatinine from the baseline values during the 24-month observation period; 5 patients in the high-normal BLB group required hemodialysis; 1 patient with high-normal and 1 with low BLB died of acute myocardial infarction; and 2 patients with high BLB were lost to followup. A total of 45 (50.6%) patients reached the primary endpoint. Only 9 (9/27, 33.3%) patients had a body lead burden <80 *μ*g, and 36 (36/62; 58.1%) of these subjects had body lead burdens >80 *μ*g (Logrank tests, *P* = 0.023) (Figure 2). In addition, GEE with linear analysis showed that BLB or BLL were significant variables for predicting the progression of eGFR, after adjusting for other variables (Tables 4 and 5). Each 1 *μ*g increase in BLB led to a decrease of 0.022 mL/min/1.73 m^2^ in eGFR (*P* = 0.009) and each 1 *μ*g/dL increase in BLL led to a 0.298 mL/min/1.73 m^2^ decrease in eGFR (*P* = 0.010) during the 2-year study period. Moreover, multivariate Cox regression analysis demonstrated that BLB was a significant risk factor (hazard ratio [HR] = 1.01, 95% confidence interval [CI]: 1.01-1.02;  *P* < 0.001) for achieving primary outcome in type II diabetic patients, even after adjustment for other factors (Table 6). Similarly, multivariate Cox regression analysis demonstrated that BLB >80 *μ*g was a significant risk factor (*HR* = 2.79, 95% CI: 1.25–6.25;  *P* = 0.012) for achieving primary outcome in these patients.

## 4. Discussion

The results of the present study indicate that BLB and BLL, even at low levels, are important risk factors for progressive diabetic nephropathy. These associations were strong, dose dependent, and consistent, even after comprehensive adjustments for other covariates. Our result is similar to those of previous reports showing that increased BLL is associated with a progressive decline in renal function in the general population [4, 5].

In comparison with our previous work [14], this study enrolled a different study cohort and showed several novel findings. First, patients with a high-normal BLB showed a higher incidence of progressive diabetic nephropathy than those with low-normal BLB, although the corresponding variables were not different between the 2 groups during the 2-year followup period. Moreover, similar results were obtained in the sensitivity test. Second, each increment of 10 *μ*g/dL of BLL was determined to potentially decrease GFR by 3.0 mL/min/1.73 m^2^ after adjustment for covariates. In addition to BLB, BLL is a strong predictor of progressive diabetic nephropathy and can be easily monitored in clinical practice. Importantly, there were no safe limits of lead indices in our study. Consistent with our results, previous studies of healthy populations indicated a high correlation between measured BLL and BLB [20, 21]. Therefore, one can assume that under conditions of constant environmental lead exposure, a higher BLL should correspond to a higher BLB. Third, the present study included a more homogenous population than our previous study [14]. Only patients with stage 3 CKD were included in the present study, whereas patients with stages 2, 3, and 4 CKD were included in our previous work [14]. Achieving primary outcome in patients with different stages of CKD is associated with confounding effects. Moreover, patients with stage 4 CKD may have hyperparathyroidism, which can cause osteopathy; increase BLB, as measured by EDTA tests [22]; and result in selection bias in the classification of high-normal or low-normal BLB groups. Fourth, the eGFR was calculated from the Chinese-modified MDRD formula for CKD patients with type II diabetes rather than the formula used for American CKD patients. Lastly, because the present study used stricter definitions of the primary outcome (a 2-fold versus a 1.5-fold increase in serum creatinine level from that of the baseline) and a longer followup period (24 months versus 12 months) than previous studies [13, 14], a more definitive conclusion regarding the long-term effect of environmental exposure to lead on progressive diabetic nephropathy can be drawn. 

The mean BLL of our patients was only 4.3 *μ*g/dL, which is lower than observed in our previous study [14] and slightly higher than that reported by nationwide surveys in Taiwan (3.0 *μ*g/dL) [23], Europe (2.57 *μ*g/dL) [24], and the USA (3.5 *μ*g/dL) [25]. This difference could be the result of the older age (mean, 60.1 years old) of our study patients. The mean BLB of our patients was only 109.9 *μ*g, which is much lower than subtle lead poisoning (>600 *μ*g) levels [7]. Although there were no differences in baseline data, Kaplan-Meier analysis showed that patients with high-normal BLB were more likely (58.1%) to achieve the primary outcome than those (33.3%) with low-normal BLB during the 24-month followup. Multivariate Cox analysis indicated each 100-*μ*g increase of BLB could lead to a 100% increase in the risk of achieving primary outcome. Consistent with this result, EDTA chelation therapy has shown benefits in retarding progressive diabetic nephropathy in type II diabetic patients with high-normal BLB [14, 26]. Hence, environmental exposure to lead may accelerate progressive diabetic nephropathy in these patients, and it is reasonable to suggest chelation therapy for patients with high-normal BLB, who accounted for 70% (62/89) of the current study patients.

 The mechanism underlying the effect of environmental exposure to low-levels of lead on accelerating the development of progressive diabetic nephropathy remains unclear. Low-level lead exposure in a rat CKD model was found to hasten progressive CKD by accelerating microvascular and tubulointerstitial injury [27]. Studies performed on animals [28, 29] have shown that chronic exposure to low-dose lead results in the generation of reactive oxygen species, reduces nitric oxide availability and the expression of angiotensin II, and increases blood pressure [29]. It also promotes hydroxyl radical generation and lipid peroxidation [30], enhances vascular reactivity to sympathetic stimulation, and decreases DNA repair capacity, which might be relevant for rapidly dividing cells in the inflamed arterial wall [31]. Moreover, chronic exposure to low-level lead-induced oxidative stress and reduced nitric oxide availability were successfully treated with a lead chelating agent or antioxidants in rats [29, 32]. These findings support that chronic exposure to low-levels of lead may have a negative effect on diabetic nephropathy. Several recent nationwide epidemiological studies also indicated that environmental exposure to lead, even at low levels, is associated with CKD in the general population [33, 34]. Moreover, higher BLL in the range below 10 *μ*g/dL was shown to be related to lower cystatin-estimated GFR [35] in adolescents. These previous studies support the current study results. However, much remains to be explored regarding the mechanisms of lead-induced progressive diabetic nephropathy.

The use of eGFR to assess altered renal function is one of the limitations of the present study. However, a study on eGFR in Chinese patients with type II diabetes conducted by Barbosa et al. [36] demonstrated a strong correlation between eGFR and isotopic GFR (*r* ^2^ = 0.95) in the Chinese population. Another limitation of this study was that BLB was not assessed using X-ray fluorescence methods. However, there are several important limitations associated with X-ray fluorescence-based methods [36], such as lack of precision, nonhomogenous lead distribution in cortical bone, and a low turnover rate with low biological activity of lead in cortical bone. However, lead that can be chelated by EDTA predominantly reflects lead concentrations in the blood and soft tissues. Because the kidneys are included among soft tissues, EDTA mobilization may reflect the lead content of the kidney [37], which may influence progressive CKD. 

## 5. Conclusion

 The results of this prospective study indicate that environmental exposure to lead may accelerate progressive diabetic nephropathy in type II diabetic patients despite the control of treatable factors during long-term followup. These results suggest that avoiding exposure to any sources of lead in the environment and chelation therapy are important in patients with BLB >80 *μ*g. The findings of the current study are important because diabetic nephropathy is the major cause of end-stage renal disease in the world. 

## Figures and Tables

**Table 1:** Baseline characteristics of patients with high-normal and low-normal body lead burden at the beginning of the observation period*.

Variable	Low-normal BLB group	High-normal BLB group	*P* value
	(*n* = 27)	(*n* = 62)	
	(*N* = 25)	(*N* = 25)	
Age (yr)			
Mean ± SD	60.7 ± 8.3	59.8 ± 10.1	0.698
Range	46–79	33–83	
Sex (no. of patients)			
Men/women	17/10	41/21	0.812^†^
Education yrs >9 yrs (no. of patients)	13 (39.4)	30 (40.5)	0.911^†^
Body-mass index (kg/m^2^)			
Mean ± SD	24.5 ± 3.0	25.1 ± 3.7	0.459
Range	17.6–32.3	14.9–33.4	
Hyperlipidemia (no. of patients) (%)^*∧*^	16 (60.6)	46 (68.9)	0.210^†^
Use of statin drugs (no. of patients) (%)	15 (57.6)	41 (60.8)	0.352^†^
Hypertension (no. of patients) (%)^*||*^	26 (97.0)	58 (94.6)	0.999^†^
Use of angiotensin-converting-enzyme inhibitors or angiotensin-receptor antagonists (no. of patients) (%)	27 (100.0)	60 (98.6)	0.999^†^
Use of nondihydropyridine calcium channel blockers (no. of patients) (%)	10 (48.5)	22 (33.8)	0.999^†^
Use of dihydropyridine calcium channel blockers (no. of patients) (%)	9 (27.3)	24 (40.5)	0.812^†^
Smoking (no. of patients) (%)	5 (15.2)	9 (24.3)	0.753^†^
History of cardiovascular disease (no. of patients) (%)	8 (36.4)	21 (29.7)	0.808^†^
Use of insulin at entry (no. of patients) (%)	6 (18.2)	16 (24.3)	0.795^†^
HbA1c (%)			
Mean ± SD	8.2 ± 2.1	8.6 ± 1.8	0.367
Range	5.8–14.0	5.7–14.7	
Mean arterial pressure (mmHg)			
Mean ± SD	96.6 ± 12.0	98.6 ± 11.9	0.477
Range	68.7–120	75–126	
Cholesterol (mg/dL)			
Mean ± SD	211.0 ± 38.2	217.2 ± 54.5	0.596
Range	124–278	105–414	
Triglycerides (mg/dL)			
Mean ± SD	211.3 ± 107.2	206.2 ± 187.6	0.697
Range	91–635	56–1185	
Serum creatinine (mg/dL)^‡^			
Mean ± SD	1.79 ± 0.21	1.88 ± 0.30	0.170
Range	1.5–3.7	1.5–3.8	
Creatinine clearance rate (mL/min/1.73 m^2^)			
Mean ± SD	44.9 ± 10.8	41.1 ± 12.0	0.123
Range	29.2–61.4	24.6–70.9	
Glomerular filtration rate^⊙^ (mL/min/1.73 m^2^)			
Mean ± SD	42.4 ± 6.2	40.8 ± 7.1	0.380
Range	31.9–58.3	30.3–59.9	
Blood lead (*μ*g/dL)^#^			
Mean ± SD	3.8 ± 3.0	4.6 ± 3.1	0.278
Range	1.6–10.4	0.8–10.3	
Body lead burden (*μ*g)^#^			
Mean ± SD	58.1 ± 16.7	132.4 ± 46.1	0.001
Range	14.4–79.8	82.8–316.8	
Daily protein excretion (g)			
Mean ± SD	2.8 ± 2.5	3.2 ± 2.4	0.364
Range	0.5–10.5	0.5–12.2	
Daily protein intake (g/kg)			
Mean ± SD	0.99 ± 0.16	0.96 ± 0.18	0.569
Range	0.60–1.55	0.53–1.68	

*A high-normal body lead burden was defined as a lead value of at least 80 *μ*g (0.39 *μ*mol) but less than 600 *μ*g (2.9 *μ*mol) and a low-normal body lead burden as a lead value less than 80 *μ*g (0.39 *μ*mol).

^†^ *P* values were calculated by Fisher's Chi-square test, except in the comparisons of age, body-mass index, serum creatinine, creatinine clearance, glomerular filtration rate, blood lead level, and body lead burden, which were calculated by Student's *t*-test.

^‡^To convert values for serum creatinine to micromoles per liter, multiply by 88.4.

^⊙^Modified equation for glomerular filtration rate of Chinese diabetic patients.

^#^To convert values for lead to micromoles per liter, multiply by 0.04286.

^*∧*^Hyperlipidemia was defined as a serum cholesterol level above 240 mg per deciliter (6.2 mmol per liter) after diet control.

^*||*^Hypertension was defined by the presence of at least two blood-pressure measurements above 140/90 mmHg in patients.

Cardiovascular diseases included ischemic heart disease, congestive heart failure, stroke, and diabetic foot.

**Table 2:** Means of renal function during the 24-month observation period (*n* = 89).

Renal function	Low-normal BLB group	High-normal BLB group	*P* (95% CI)
(mL/min/1.73 m^2^)	(*n* = 27)	(*n* = 62)	
Month 0			
Ccr	44.9 ± 10.8	41.1 ± 12.0	0.123 (−9.1–1.0)
D-GFR	42.4 ± 6.2	40.8 ± 7.1	0.300 (−1.5–4.8)
Month 6	(*n* = 27)	(*n* = 62)	
Ccr	41.5 ± 15.2	33.1 ± 13.0	0.010 (−14.6–−2.1)
D-GFR	38.1 ± 9.2	32.9 ± 8.3	0.010 (−9.1–−1.3)
Month 12	(*n* = 27)	(*n* = 62)	
Ccr	41.0 ± 19.2	26.2 ± 12.2	<0.001 (−21.6–−8.1)
D-GFR	36.6 ± 9.5	27.2 ± 8.9	<0.001 (−13.6–−5.2)
Month 18	(*n* = 26)	(*n* = 59)	
Ccr	33.1 ± 13.7	24.5 ± 10.8	0.003 (−14.1–−3.0)
D-GFR	32.7 ± 11.2	23.7 ± 8.0	<0.001 (−13.3–−4.7)
Month 24	(*n* = 24)	(*n* = 55)	
Ccr	34.2 ± 17.3	19.8 ± 9.8	<0.001 (−20.5–−8.2)
D-GFR	31.4 ± 11.2	20.2 ± 7.0	<0.001 (−15.3–−7.1)
Total decrease of renal function (mL/min/1.73 m^2^) during the 2-year observation period			
Ccr	12.1 ± 15.5	21.2 ± 8.7	0.002 (3.7–14.5)^#^
D-GFR	11.3 ± 11.7	20.5 ± 7.7	0.001 (4.8–13.7)^#^

Data were measured by the Student's *t*-test except ^#^ data by Mann-Whitney method. *P* < 0.05 means significant differences. Ccr: creatinine clearance; D-GFR: estimated GFR for Chinese patients with type II diabetes.

**Table 3:** Sensitivity analysis of renal function from month 18 to month 24 of the observation period (*n* = 89).

Renal function	Low BLB group	High BLB group	*P* (95% CI)
(mL/min/1.73 m^2^)	(*n* = 27)	(*n* = 62)	
Month 18			
Ccr	32.8 ± 13.6	24.9 ± 10.7	0.005 (−13.2–−2.5)
D-GFR	32.4 ± 11.1	24.2 ± 8.1	<0.001 (−12.4–−4.1)
Month 24			
Ccr	32.6 ± 16.1	21.5 ± 9.8	<0.001 (−16.9–−5.4)
D-GFR	30.2 ± 11.1	21.5 ± 7.5	<0.001 (−12.7–−4.7)
Total decrease of renal function (mL/min/1.73 m^2^) during the 2-year observation period			
Ccr	12.2 ± 14.6	18.6 ± 11.2	0.010 (0.7–12.0)^#^
D-GFR	12.3 ± 11.5	19.3 ± 8.4	0.006 (2.7–11.3)^#^

Data were measured by the Student's *t*-test except ^#^ data by Mann-Whitney method. *P* < 0.05 means significant differences. Ccr: creatinine clearance; D-GFR: estimated GFR for Chinese patients with type II diabetes.

**Table 4:** Longitudinal multivariate analysis of body lead burden and other predictors of progressive change in the estimated glomerular filtration rate (D-GFR), using generalized estimating equations, during the 24-month longitudinal study period (*n* = 89).

Variable	Estimate (interactive effect)*	*P* value
Age (each increment of 1 yr)	−0.271	<0.001
Gender (female versus male)	−3.575	<0.001
Smoking (no versus yes)	−0.259	0.813
Body-mass index (each increment of 1 kg/m^2^)	0.020	0.852
History of cardiovascular diseases (no versus yes)	−0.375	0.686
MAP (mmHg) (each increment of 1 mmHg)	− 0.054	0.033
Cholesterol (mg/dL) (each increment of 1 mg/dL)	−0.002	0.811
Triglycerides (mg/dL) (each increment of 1 mg/dL)	− 0.004	0.075
HbA1c (%) (each increment of 1%)	0.013	0.793
Serum creatinine (mg/dL) (each increment of 1 mg/dL)	−6.997	<0.001
Body lead burden (*μ*g) (each increment of 1 *μ*g)	−0.022	0.009
Daily protein intake (g/kg) (each increment of 1 g/kg)	1.287	0.460
Daily protein excretion (g) (each increment of 1 g)	−0.417	0.035

The interactive effect of variables was calculated by a generalized estimating equation. Negative values for the interactive effect indicate a decline in the glomerular filtration rate, and positive values indicate an increase. Cardiovascular diseases included ischemic heart disease, congestive heart failure, stroke, and diabetic foot. MAP: mean arterial pressure.

**Table 5:** Longitudinal multivariate analysis of blood lead level and other predictors of progressive change in the estimated glomerular filtration rate (D-GFR), using generalized estimating equations, during the 24-month longitudinal study period (*n* = 89).

Variable	Estimate (interactive effect)*	*P* value
Age (each increment of 1 yr)	−0.268	<0.001
Gender (female versus male)	−3.261	<0.001
Smoking (no versus yes)	−0.631	0.604
Body-mass index (each increment of 1 kg/m^2^)	−0.018	0.861
Previous cardiovascular diseases (no versus yes)	−0.220	0.818
MAP (mmHg) (each increment of 1 mmHg)	− 0.057	0.025
Cholesterol (mg/dL) (each increment of 1 mg/dL)	−0.006	0.406
Triglycerides (mg/dL) (each increment of 1 mg/dL)	− 0.005	0.024
HbA1c (%) (each increment of 1%)	−0.002	0.976
Serum creatinine (mg/dL) (each increment of 1 mg/dL)	−7.550	<0.001
Blood lead level (*μ*g/dL) (each increment of 1 *μ*g/dL)	−0.298	0.010
Daily protein intake (g/kg) (each increment of 1 g/kg)	1.143	0.539
Daily protein excretion (g) (each increment of 1 g)	−0.400	0.045

The interactive effect of variables was calculated by a generalized estimating equation. Negative values for the interactive effect indicate a decline in the glomerular filtration rate, and positive values indicate an increase. Cardiovascular diseases included ischemic heart disease, congestive heart failure, stroke, and diabetic foot. MAP: mean arterial pressure.

**Table 6:** Cox regression analysis of the overall risk of the primary outcome of progressive renal insufficiency, according to baseline prognostic factors (*N* = 89).

Variable	Hazard ratio (95% CI)*	*P* value
Age (each increment of 1 yr)	0.98 (0.94–1.02)	0.337
Female sex	1.83 (0.87–3.83)	0.111
Smoking (no versus yes)	0.75 (0.26–2.12)	0.582
Baseline body-mass index (each increment of 1 kg/m^2^)	0.90 (0.81–0.99)	0.023
Previous cardiovascular diseases (no versus yes)	0.55 (0.24–1.29)	0.170
MAP (mmHg) (each increment of 1 mmHg)	1.03 (1.00–1.06)	0.088
Cholesterol (mg/dL) (each increment of 1 mg/dL)	1.00 (0.99–1.01)	0.822
Triglycerides (mg/dL) (each increment of 1 mg/dL)	1.00 (1.00-1.00)	0.937
HbA1c (%) (each increment of 1%)	0.98 (0.83–1.16)	0.806
Baseline serum creatinine (each increment of 1 mg/dL)	0.29 (0.06–1.29)	0.104
Body lead burden (each increment of 1 *μ*g)	1.01 (1.01–1.02)	<0.001
Baseline daily protein intake (each increment of 1 g/kg)	0.41 (1.06–1.44)	0.462
Baseline daily protein excretion (each increment of 1 g)	1.24 (1.12–1.42)	0.008

Cardiovascular diseases included ischemic heart disease, congestive heart failure, stroke, and diabetic foot. MAP: mean arterial pressure.
